# BRIDGING SOCIAL GAPS WITH THE VIRTUAL FRIENDLY VISITORS INITIATIVE

**DOI:** 10.1093/geroni/igae098.2859

**Published:** 2024-12-31

**Authors:** Barbara Gordon, Pamela Yankeelov, Anna Faul, Chelsea Miceli, Samantha Cotton

**Affiliations:** University of Louisvlle, Louisville, Kentucky, United States; University of Louisvlle, Louisville, Kentucky, United States; University of Louisville, Louisville, Kentucky, United States; University of Louisville, Louisville, Kentucky, United States; University of Louisville, Louisville, Kentucky, United States

## Abstract

Social isolation affects one in four older adults aged 65 and over, significantly increasing the risk of premature death and dementia. The COVID-19 pandemic exacerbated this isolation, highlighting the urgent need for strategies to foster connections among older adults. Utilizing COVID-Cares Act funds, our Geriatrics Workforce Enhancement Program (GWEP) transformed the Friendly Visitors Program into a virtual initiative (VFVP), distributing tablets to enable social engagement through virtual visits. Ninety-six older adults actively engaged with 45 volunteer visitors across 601 virtual visits. The areas of interaction revolved around life reviews, family support, validating feelings, social determinants of health and hobbies. Evaluation data indicates improved mood, increased awareness of community resources, and high levels of satisfaction with the program. These visits led to the identification of changes in biological and behavioral health, home health needs, and neglect concerns by the visitors, resulting in appropriate referrals. VFVP not only improved mood and social engagement among participating older adults but also played a crucial role in monitoring health, connecting participants with vital resources, and assisting with environmental challenges to support independence. This success underscores the importance of community and technology in combating isolation and promoting the well-being of older adults, highlighting the potential of virtual programs. In this presentation, our team will explore the critical role friendly visitors play in monitoring health conditions. The session will also examine how friendly visitors can act as vital links between older adults and essential community resources, ensuring that needs ranging from nutrition to mobility are met.

